# Errata

**Published:** 2014-01-10

## Vol. 60, No. 53

In the “Summary of Notifiable Diseases, United States, 2011,” on page 26, in Table 1, the row for “Ehrlichiosis/Anaplasmosis” should have been left blank, with no values. Three rows down, above “Undetermined,” the row for “*Anaplasma phagocytophilum*” should have been inserted with all of the values that were incorrectly listed for “Ehrlichiosis/Anaplasmosis.”

In addition, on page 41, in Table 3, the row for “Ehrlichiosis/Anaplasmosis” should have been left blank, with no values. Three rows down, above “Undetermined,” the row “*Anaplasma phagocytophilum*” should have been inserted with all of the values that were incorrectly listed for “Ehrlichiosis/Anaplasmosis.”

Finally, on page 43, in Table 4, the diseases are presented out of order. The order should be as follows: *Anaplasma phagocytophilum*, *Ehrlichia chaffeensis*, *Ehrlichia ewingii*, and Undetermined. The total counts are in correct order.

## Vol. 62, No. 33

In the report, “Final 2012 Reports of Nationally Notifiable Diseases,” in Table 2, multiple errors occurred. On page 671, for Babesiosis, United States, the values for Total, Confirmed, and Probable should read **937**, **716**, and **221**, respectively. For Babesiosis, S. Atlantic, the values for Total, Confirmed, and Probable should read **3**, **1**, and **2**, respectively. For Babesiosis, District of Columbia, the entries for Total, Confirmed, and Probable all should read **N**. On page 679, for Rabies, Animal, the United States value should read **4,541**; the E.N. Central value should read **170**, and the Illinois value should read **63**.

On page 662, the bottom section of Table 2 should appear as follows:

**Table:** 

Area	*Chlamydia trachomatis* infection^†^	Cholera	Coccidioidomycosis	Cryptosporidiosis			Cyclosporiasis

				Total	Confirmed	Probable	
**Territories**							
American Samoa	—	—	N	N	N	N	N
C.N.M.I.	—	—	—	—	—	—	—
Guam	1,031	—	—	—	—	—	—
Puerto Rico	6,227	—	N	N	N	N	N
U.S. Virgin Islands	802	—	—	—	—	—	—

## Vol. 62, Nos. 51 & 52

In the second announcement on page 1052, an error occurred in the first sentence of the second paragraph. That sentence should read, “January **5–11**, 2014, is National Folic Acid Awareness Week.

